# Nurses’ Knowledge and Attitudes about Adult Post-Operative Pain Assessment and Management: Cross Sectional Study in Qatar

**DOI:** 10.3390/nursrep14030153

**Published:** 2024-08-21

**Authors:** Haya Samara, Lily O’Hara, Kalpana Singh

**Affiliations:** 1 Nursing Education Department, Hamad Medical Corporation, Doha 122014, Qatar; 2 Department of Public Health, QU Health, Qatar University, Doha P.O. Box 2713, Qatar; lohara@qu.edu.qa; 3 Department of Nursing Research, Hamad Medical Corporation, Doha 122014, Qatar; nkalpanasingh@gmail.com

**Keywords:** knowledge, attitudes, adult post-operative, pain assessment, pain management, Qatar, nurses

## Abstract

Background: Pain is a complex and challenging phenomenon. People have different pain experiences, but everyone has the right to effective pain management. Pain assessment and management are integral components of a nurse’s role. Aim: To assess the knowledge and attitudes of nurses in Qatar about adult post-operative patients’ pain assessment and management, and the factors that may be associated with such knowledge and attitudes. Methods: Post-operative registered nurses from all peri-operative areas at Hamad Medical Corporation participated in a cross-sectional online survey using a self-administered questionnaire. A knowledge and attitudes (K&A) score was calculated. Associations between K&A and potential explanatory variables were assessed using *t*-tests and one-way ANOVA. Results: A total of 151 post-operative nurses participated in the study. The mean knowledge and attitudes (K&A) score was 19.6 ± 4.5 out of 41 (48%), indicating a large deficit in nurses’ knowledge and attitudes about adult post-operative pain. There were no statistically significant differences in the mean K&A scores of participants based on gender, nationality, education level, marital status, workplace facility, current job designation, or hours of pain education. Conclusions: There is a significant deficit in post-operative nurses’ knowledge and attitudes about pain across the nursing workforce in post-operative care. Implications for nursing education and policy: Evidence-based, innovative nursing education courses are needed to improve nurses’ knowledge and attitudes about pain assessment and management. Health service policy is required to ensure that evidence-based in-service education on pain management is compulsory for all nurses. This study was not registered.

## 1. Introduction

Pain has been described as a complex and challenging phenomenon. The care of patients suffering from pain is a difficult nursing task that requires a high level of knowledge, competent intervention, and a genuine belief in what patients report [1]. Worldwide, pain is considered a major issue. People experience pain acutely, chronically, or in combination [2,3]. Acute pain is short-term, and chronic pain lasts more than three months. It is unknown precisely what contributes to individual cases of acute pain [3], and treatment is therefore more difficult [4]. One in five adults worldwide will suffer from acute pain each year and one in ten adults has chronic pain [5]. Pain can be devastating and can affect and be affected by a person’s state of mind [6]. Short- and long-term consequences of pain include depression, work loss, and impaired social relationships [2,7].

Between 50% and 80% of patients report pain from procedures, surgery, or diseases during hospitalization [8,9]. In a post-operative situation, recent onset of pain and limited pain duration are usually evident [10]. Fifty percent of post-operative patients complain of pain within 24 h of surgery, which decreases slowly over several days if not properly assessed and managed [2,7]. In a 2016 cross-sectional retrospective study of over 15,000 surgery patients in the United Kingdom, 11% reported extreme pain and 37% complained of moderate pain in the first 24 h after surgery [11].

Caring for a patient with pain is a challenging task that requires up-to-date knowledge, skilled interventions, and an attitude that ensures trust, care, and a genuine belief in what the patient says and reports [1]. Despite the latest advances in medical and nursing care, some patients cannot accurately describe their pain [1]. Accurate assessment of pain is the foundation of effective pain management. Nurses must be knowledgeable about pain assessment and should not hold harmful opinions and beliefs about pain management [12]. According to an ethnographic study by Yassin et al. [13], nurses’ lack of knowledge about pain and negative attitudes towards pain result in unacceptable delays in pain intervention. The study’s key results showed that there is a socially orchestrated mechanism of pain management delays built into nurses’ work.

Nurses’ negative attitudes and lack of knowledge about pain management are major impediments to prompt and effective pain management [14,15]. Nurses’ attitudes strongly influence their pain assessment [16]. Knowledge and attitudes (K&A) about pain assessment and management are lacking [15,17]. Up to 60% of nurses have insufficient knowledge regarding the use of pain tools [10]. Studies conducted in many countries have identified gaps in nurses’ knowledge and attitudes about pain assessment and management [14,17,18,19,20,21,22].

Nurses’ knowledge about pain assessment and management is based on education, and personal and professional experiences. Nurses’ attitudes about pain assessment and management are shaped by their values, cultural beliefs, social groups, religion, lived experiences, age, and gender [23]. Professional attitudes can be learned from the clinical environment, preceptorship, mentorship, and nurses imitating their peers. In order to ensure patient care and wellbeing, including in the post-operative setting, it is vital to understand nurses’ level of knowledge and attitudes towards pain. Various methods and tools have been used to investigate nurses’ knowledge and attitudes to pain. Although qualitative studies have been conducted on nurses’ knowledge and attitudes towards pain in cancer care in Qatar [13,24], to date, there are no quantitative studies of nurses’ knowledge and attitudes in the post-operative setting in Qatar.

The Knowledge, Attitudes, and Practice (KAP) model is a widely used framework for assessing and understanding behaviors related to health and social issues [25]. It has been applied to various topics, including pain management, and provides a valuable framework for investigating nurses’ knowledge, attitudes, and practices in pain assessment and management. This information can then be used to identify areas of strength and weakness and to develop professional development initiatives to improve pain assessment and management practices [25].

The aim of this study was to address the gap in the literature by investigating the pain-related knowledge and attitudes of nurses in Qatar about adult post-operative patients’ pain assessment and management, and the factors that may be associated with such knowledge and attitudes. The research questions were as follows: 1. what are the levels of nurses’ knowledge and attitudes about adult post-operative patients’ pain assessment and management; 2. what are the relationships between nurses’ knowledge and attitudes about adult post-operative patients’ pain assessment and management and sociodemographic factors; and 3. what is the relationship between previous attendance at pain education programs and the level of knowledge and attitudes about adult post-operative patients’ pain assessment and management.

## 2. Methods

### 2.1. Research Design

The study was a cross-sectional online survey using a self-administered questionnaire. Data were collected from November 2020 to January 2021. Strengthening the Reporting of Observational Studies in Epidemiology (STROBE) guidelines were used to ensure comprehensive reporting of the study and findings [26].

### 2.2. Sample, Setting, and Participants

The study involved a universal sample of registered nurses working in Hamad Medical Corporation (HMC) hospitals in Qatar, who provided bedside care for adult patients in post-operative care areas.

The Knowledge and Attitudes Survey Regarding Pain tool (KASRP) [27] was used to collect data. KASRP is a widely-used self-administered questionnaire to evaluate nurses’ knowledge on pain in six areas: medication, assessment, intervention, addiction, spiritual/cultural and pathophysiology. The reliability of the KASRP was established (*r* > 0.80) by repeat testing in a continuing education class of staff nurses (*n* = 60) [27]. Internal consistency has been established (Cronbach’s α > 0.70) for both knowledge and attitude domains [27]. A review of the tool by pain experts confirmed the content validity. The tool content is derived from standards of pain management such as the American Pain Society, the World Health Organization, and the National Comprehensive Cancer Network Pain Guidelines [27]. Construct validity was established by comparing scores of nurses at various levels of expertise such as new graduates, oncology nurses, students, junior and senior pain experts. The KASRP is freely available online [27].

The instrument has 41 items including 22 true/false statements, 15 multiple choice questions, and two scenarios about patient care, each with two questions about pain assessment and appropriate pain treatment. Correct answers are given a score of 1, and incorrect or unanswered items are given a score of 0. The total score, known as the K&A score, ranges between 0 and 41, with higher scores indicating a higher level of pain-related knowledge and positive attitudes. For ease of interpretation and comparison, the score is converted to a percentage, and 80% is regarded as the minimum satisfactory score [27].

The original version of KASRP was administered in English, as English is the official communication language in HMC. All nurses must speak English, and as such, the English language KASRP was considered appropriate without the need for translation. Prior to implementation, the instrument was pilot tested on 10% of the sample to ensure that members of the study population understood the items clearly. The instrument has not been validated in Qatar.

### 2.3. Ethical Considerations

The study was conducted in full conformance with the principles of the Declaration of Helsinki, Good Clinical Practice, and within the Ministry of Public Health laws and regulations in Qatar. The study was approved by the HMC Medical Research Committee approval number (MRC-01-20-796-DSA) and Qatar University Institutional Review Board (QU-IRB) (Approval number QU-IRB 1451-EA/21).

### 2.4. Data Analysis Method

Descriptive statistics were used to describe the characteristics of the participants, including frequencies and percentages for categorical variables and means, ranges, and standard deviations for continuous variables. Approximately 22% of the item responses were missing. For the analysis, complete case analysis was undertaken. Descriptive statistics were used to summarize the percentage of correct responses for each item in the KASRP, and the K&A score mean and standard deviation. Independent *t*-tests and One-way Analysis of Variance (ANOVA) were employed to analyze the associations between the K&A score, and the sociodemographic variables and the number of hours spent in pain education. Data were entered and analyzed with Stata 15.1 statistical software (StataCorp. 2017. Stata Statistical Software: Release 15. StataCorp LLC: College Station, TX, USA).

## 3. Results

A total of 151 post-operative nurses responded to the survey. The majority of participants were female (70%), married (71%), and non-Qatari (97%), of which 42% were Filipino, and 29% were Indian. The mean age was 37 ± 7.8 years, and the mean years of experience as a nurse was 13.5 ± 7.1 years. Most participants were staff nurses (76%), had a bachelor’s degree (77%), and received one to two hours (37%) or three to five hours (27%) of pain education in the last two years. Over half the participants worked at the two major hospitals, Hamad General Hospital and Hazm Mebaireek General Hospital (Table 1).

The first research question for the study asked what are the levels of nurses’ knowledge and attitudes about adult post-operative patients’ pain assessment and management? The proportion of participants correctly answering each item in the KASRP is shown in Table 2. The mean K&A score was 19.6 ± 4.5 out of 41 (48%) with a range of 8 to 32 (19.5% to 78.0%). No participants scored above the pass mark of 80%. Although 60% of participants correctly identified that patients should not be encouraged to endure pain before using an opioid, less than a quarter correctly identified that if the source of the patient’s pain is unknown, opioids should not be used during the pain evaluation period, as this could reduce the ability to correctly diagnose the cause of pain. Less than one in five participants correctly identified the most likely reason a patient may require pain medication. More than half the participants incorrectly encourage their patients to tolerate the pain before giving them any pain medications. Most participants failed to identify the correct pain score if the patient’s vital signs are stable and their facial expressions are relaxed. Less than half the participants correctly identified the conversion of oral morphine doses or identified the use of sterile water (placebo injection) as a helpful way to determine if the patient’s pain is real.

The second research question asked what are the relationships between nurses’ knowledge and attitudes about adult post-operative patients’ pain assessment and management and sociodemographic factors? No statistically significant relationships were found between K&A score and age, education level, marital status, facility, or nurse’s designation (Table 3). The third research question asked what is the relationship between previous attendance at pain education programs and the level of knowledge and attitudes about adult post-operative patients’ pain assessment and management? There was no statistically significant relationship between the K&A score and the number of hours spent in pain education (Table 3).

## 4. Discussion

Nurses in this study had limited knowledge of pain assessment and management, and poor attitudes towards pain assessment and management. The average K&A score was 47.8% and no participants scored at or above the pass mark of 80% [27]. Although these results are poor and cause for concern, they are consistent with the results of a recent systematic review and meta-analysis of 18 studies involving 7942 nurses, which found that the average K&A score was 52.9% (95% CI: 47.2–58.6) [28]. None of the studies in the meta-analysis had average scores at or above the 80% pass mark. Similar or lower mean scores to those in this study have been found in Sudan, mean score 19% [12], Jordan 41% [29], 43% [30], 45% [31], 48% [32], 34% [33], Saudi Arabia 42% [34], 46% [17], China 40% [35] Turkey 40% [36], Palestine 45% [37], Vietnam 45% [38], and Eritrea 49.5% [39]. Nurses who score lower than 80% have lower levels of ability to effectively and adequately care for patients complaining of pain [19,20,23,36,40,41].

This study found no correlation between nurses’ pain knowledge and attitudes and any potential predictor variables in this study. This finding was largely consistent with the meta-analysis of 18 studies, which found that K&A scores were not associated with age, sex, or professional years of experience [28]. Similar to our study, Al Qadire and Al Khalaileh also found no association between K&A scores and pain education [32]. In contrast, other studies [35,42,43] and a meta-analysis found that K&A scores were associated with previous pain education in 11 out of 18 studies [28].

In this study, K&A scores were not significantly associated with years of experience. This is consistent with the findings across 18 studies which found that years of experience was poorly associated with K&A [28]. In contrast, Al-Shaer et al. found that nurses who worked for 16 years or more in their unit scored significantly higher K&A scores than nurses who worked for one to five years in their units [19,44]

This study found no association between K&A scores and level of education, consistent with the meta-analysis [28], a study on nurses working in a cancer care centre in Qatar [24], and studies on nurses in Jordan [45], Italy [41] and the USA [19,44]. In contrast, studies on nurses in Jordan [32], Palestine [37], Iceland [46], USA [23], Canada [47], and Turkey [36] found that higher levels of education were associated with higher K&A scores.

This study found a low level of knowledge about pharmacological interventions, particularly regarding the appropriate opioid selection, dosing, and converting between different types of opioids and the assessment/reassessment of pain after opioid administration. This was consistent with other studies that found that pain management pharmacology is the weakest area [30,47,48], and the systematic review that found that intervention had the lowest score across the six domains [28]. Addiction and opioid overdose are considered one of the most common opioid-related fears for nurses [49]. To address these fears, effective strategies such as frequent pain assessment should be employed by nurses to help them understand the effects of opioids and their side effects. Current pain management strategies involve taking a proactive approach to preventing pain rather than a reactive approach to treating it [50]. Patients are encouraged to speak about their pain, and nurses are encouraged to thoroughly assess and reassess pain. Treatment is customized based on patients’ physiologic and psychologic need for analgesics so that effective pain control through efficient pain assessment is achieved. A patient who receives opioids should be constantly evaluated. Equally important is clear and effective communication between all healthcare providers, particularly between pharmacists and medical teams, who are privileged to prescribe pain medication. By applying such strategies, adequate pain management will be ensured.

In this study, almost 60% of nurses incorrectly believed that vital signs are always reliable indicators of the intensity of a patient’s pain. This finding is similar to other studies [1,51]. Nonverbal cues and behavioral manifestations are crucial indicators of pain [1]. However, only 26% of participants in this study correctly identified that patients can sleep even if they have severe pain. One of the highest areas of concern was related to nurses’ beliefs about the most likely reason a patient complaining of pain would request increased doses of pain medication. Only 18% of participants correctly identified vital signs and facial expressions as manifestation of behavioral cues to pain.

Overall, the study found a significant deficit in post-operative nurses’ knowledge and attitudes regarding pain assessment and management. These results reinforce the need for well-structured, tailored educational programs to improve nurses’ pain knowledge and attitudes. Continuous educational programs support nurses to develop theoretical and clinical knowledge that improves practice [28,52,53]. For such programs to be successful, barriers and enablers of effective pain assessment and management must be addressed. Barriers include nurse workload, and factors related to the patient such as instability, inability to communicate, and sedation interfering with pain assessment [54]. Enablers include the nursing unit prioritizing pain assessment and management, having enthusiastic and motivated staff, and the use of protocols, guidelines, and standardized assessment tools [54].

## 5. Strengths and Limitations

The major limitation of this cross-sectional study is the inability to demonstrate causal links. The findings are particular to HMC post-operative nurses and cannot be applied to all nurses working in Qatar or other countries. The length of the instrument and the time constraints faced by nurses may be one reason for some of the KASRP’s missing responses. Nurses’ replies to the question concerning the number of hours spent on pain education during the previous two years may have been influenced by recall bias. A strength of this study is that the instrument was simple to complete and therefore presented a relatively low burden for busy nurses. Based on the estimated number of nurses working in post-operative care, the response rate was 100%. This unusual finding may have been due to an error in the estimation of the number of nurses in post-operative care provided by the peri-operative nursing network. The true number of nurses may have been greater. Alternately, the number may be accurate, and the response rate was so high due to the reminders sent and the support of the nurse managers. If this is the case, the findings are likely to be an accurate representation of the true state of pain-related K&A in post-operative nurses in HMC.

## 6. Conclusions

Given the very low level of knowledge and attitudes found in this study, exploratory qualitative research is required on nurses’ perceptions about the facilitators and barriers that may influence their knowledge, attitudes, and practice in post-operative pain assessment and management. Building on this, implementation research is required to develop and test educational strategies to address the most significant deficits. Culturally sensitive nursing education should be tailored to nurses’ characteristics and settings and include topics such as pain symptoms; pain assessment; analgesic mechanisms; dosage and duration and the effects and side effects of painkillers; analgesic evaluation; and non-pharmacological pain management. Innovative teaching strategies such as case studies or simulations should be used, and courses should be updated frequently. Health service policies are required to ensure the participation of nurses in pain management education programs. This will require institutional culture changes to make pain management a top priority for nurses.

## Figures and Tables

**Table 1:** Characteristics of the study participants.

Characteristic		Total *N* = 151 (%)
Gender	Female	105 (69.5%)
	Male	46 (30.5%)
Age in years mean (SD)		37.0 (7.8)
Education (highest level achieved)	Bachelor	116 (76.8%)
	Diploma	19 (12.6%)
	Master	16 (10.6%)
Years of experience mean (SD)		13.5 (7.1)
Marital status	Divorced	3 (2.0%)
	Married	106 (70.7%)
	Single	40 (26.7%)
	Widow	1 (0.7%)
Nationality	Non-Qatari	147 (97.4%)
	Indian	42 (28.57%)
	Filipino	62 (42.18%)
	Others	43 (26.65%)
	Qatari	4 (2.6%)
Number of hours of pain education in the last 2 years	0 h	8 (5.3%)
	1–2 h	56 (37.1%)
	3–5 h	40 (26.5%)
	6–8 h	22 (14.6%)
	9 h or more	25 (16.6%)
Facility	ACC	22 (14.6%)
	AKH	5 (3.3%)
	AWH	14 (9.3%)
	HGH	39 (25.8%)
	HMGH	41 (27.2%)
	TCH	6 (4.0%)
	WWRC	24 (15.9%)
Job designation	Acting Charge Nurse	1 (0.7%)
	Charge Nurse	15 (9.9%)
	Director of Nursing	1 (0.7%)
	Head Nurse	11 (7.3%)
	Nurse Educator	5 (3.3%)
	Patient Care Assistant	3 (2%)
	Staff Nurse	115 (76.2%)

Legend: SD = Standard Deviation, ACC = Ambulatory Care Center, AKH = Al Khor Hospital, AWH = Al Wakra Hospital, HGH = Hamad General Hospital, HMGH = Hazm Mebaireek General Hospital, TCH = The Cuban Hospital, WWRC = Women’s Wellness and Research Center.

**Table 2:** Percentage of Correct Responses for Items of the Knowledge and Attitudes Survey Regarding Pain Among Nurses.

Questions	Correct Responses *n* (%)
Vital signs are always reliable indicators of the intensity of a patient’s pain.	48 (40.7%)
2. Because their nervous system is underdeveloped, children under two years of age have decreased pain sensitivity and limited memory of painful experiences.	60 (50.8%)
3. Patients who can be distracted from pain usually do not have severe pain.	51 (43.2%)
4. Patients may sleep in spite of severe pain.	31 (26.3%)
5. Aspirin and other nonsteroidal anti-inflammatory agents are NOT effective analgesics for painful bone metastases.	40 (33.9%)
6. Respiratory depression rarely occurs in patients who have been receiving stable doses of opioids over a period of months.	71 (60.2%)
7. Combining analgesics that work by different mechanisms (e.g., combining an NSAID with an opioid) may result in better pain control with fewer side effects than using a single analgesic agent.	100 (84.7%)
8. The usual duration of analgesia of 1–2 mg morphine IV is 4–5 h.	58 (49.2%)
9. Opioids should not be used in patients with a history of substance abuse.	37 (31.4%)
10. Elderly patients cannot tolerate opioids for pain relief.	91 (77.1%)
11. Patients should be encouraged to endure as much pain as possible before using an opioid.	71 (60.2%)
12. Children less than 11 years old cannot reliably report pain so clinicians should rely solely on the parent’s assessment of the child’s pain intensity.	81 (68.6%)
13. Patients’ spiritual beliefs may lead them to think pain and suffering are necessary.	87 (73.7%)
14. After an initial dose of opioid analgesic is given, subsequent doses should be adjusted in accordance with the individual patient’s response.	109 (92.4%)
15. Giving patients sterile water by injection (placebo) is a useful test to determine if the pain is real.	55 (46.6%)
16. Vicodin (hydrocodone 5 mg + acetaminophen 300 mg) PO is approximately equal to 5–10 mg of morphine PO.	63 (53.4%)
17. If the source of the patient’s pain is unknown, opioids should not be used during the pain evaluation period, as this could mask the ability to correctly diagnose the cause of pain.	27 (22.9%)
18. Anticonvulsant drugs such as gabapentin (Neurontin) produce optimal pain relief after a single dose.	52 (44.1%)
19. Benzodiazepines are not effective pain relievers and are rarely recommended as part of an analgesic regiment.	78 (66.1%)
20. Narcotic/opioid addiction is defined as a chronic neurobiologic disease, characterized by behaviors that include one or more of the following: impaired control over drug use, compulsive use, continued use despite harm, and craving.	110 (93.2%)
21. The term ‘equianalgesia’ means approximately equal analgesia and is used when referring to the doses of various analgesics that provide approximately the same amount of pain relief.	107 (90.7%)
22. Sedation assessment is recommended during opioid pain management because excessive sedation precedes opioid-induced respiratory depression.	109 (92.4%)
23. The recommended route of administration of opioid analgesics for patients with persistent cancer-related pain is:	24 (23.3%)
24. The recommended route of administration of opioid analgesics for patients with brief, severe pain of sudden onset such as trauma or post-operative pain is:	12 (11.7%)
25. Which of the following analgesic medications is considered the drug of choice for the treatment of prolonged moderate to severe pain for cancer patients?	71 (68.9%)
26. A 30 mg dose of oral morphine is approximately equivalent to:	43 (41.7%)
27. Analgesics for post-operative pain should initially be given:	58 (56.3%)
28. A patient with persistent cancer pain has been receiving daily opioid analgesics for 2 months. Yesterday the patient was receiving morphine 200 mg/hour intravenously. Today he has been receiving 250 mg/hour intravenously. The likelihood of the patient developing clinically significant respiratory depression in the absence of new comorbidity is:	12 (11.7%)
29. The most likely reason a patient with pain would request increased doses of pain medication is:	18 (17.5%)
30. Which of the following is useful for treatment of cancer pain?	63 (61.2%)
31. The most accurate judge of the intensity of the patient’s pain is:	79 (76.7%)
32. Which of the following describes the best approach for cultural considerations in caring for patients in pain?	64 (62.1%)
33. How likely is it that patients who develop pain already have an alcohol and/or drug abuse problem?	58 (56.3%)
34. The time to peak effect for morphine given IV is:	77 (74.8%)
35. The time to peak effect for morphine given orally is:	58 (56.3%)
36. Following abrupt discontinuation of an opioid, physical dependence is manifested by the following	16 (15.5%)
37. Which statement is true regarding opioid induced respiratory depression?	60 (58.3%)
38.A. On the patient’s record you must mark his pain on the scale below. Select the number that represents your assessment of Andrew’s pain.	21 (20%)
38.B. Your assessment above was made two hours after Andrew received morphine 2 mg IV. Half hourly pain ratings following the injection ranged from 6 to 8 and he had no clinically significant respiratory depression, sedation, or other untoward side effects. He has identified 2/10 as an acceptable level of pain relief. His physician’s order for analgesia is “morphine IV 1–3 mg q1h PRN pain relief.” What action will you take at this time?	8 (7%)
39.A. On the patient’s record you must mark his pain on the scale below. Select the number that represents your assessment of Robert’s pain:	19 (18%)
39.B. Your assessment above is made two hours after Robert received morphine 2 mg IV. Half hourly pain ratings following the injection ranged from 6 to 8 and he had no clinically significant respiratory depression, sedation, or other untoward side effects. He has identified 2/10 as an acceptable level of pain relief. His physician’s order for analgesia is “morphine IV 1–3 mg q1h PRN pain relief.” What action will you take at this time?	17 (17%)

**Table 3:** Association between knowledge and attitude scores and demographic characteristics.

	N	Knowledge and Attitude Score Mean (SD)	*p*-Value
Age			
25–45 years	130	19.6 (4.6)	0.8
46–60 years	19	19.3 (4.3)	
Gender			
Female	105	19.1 (4.4)	0.068
Male	46	20.7 (4.7)	
Education			
Bachelor	116	19.9 (4.1)	0.13
Diploma	19	17.4 (4.6)	
Master	16	20.3 (6.8)	
Marital Status			
Single	44	19.9 (4.6)	0.59
Married	106	19.4 (4.5)	
Facility			
ACC	22	19.7 (4.3)	0.56
AKH	5	16.5 (5.9)	
AWH	14	21.4 (5.0)	
HGH	39	19.4 (4.9)	
HMGH	41	20.0 (4.3)	
TCH	6	17.3 (4.5)	
WWRC	24	19.8 (4.1)	
Hours of pain education			
0 h	8	16.8 (4.0)	0.4
1–2 h	56	18.9 (5.0)	
3–5 h	40	20.2 (4.4)	
6–8 h	22	19.6 (4.7)	
9 h or more	25	20.6 (3.3)	
Nationality			
Non-Qatari	147	19.7 (4.5)	0.056
Qatari	4	14.7 (0.6)	

Legend: ACC = Ambulatory Care Center, AKH = Al Khor Hospital, AWH = Al Wakra Hospital, HGH = Hamad General Hospital, HMGH = Hazm Mebaireek General Hospital, TCH = The Cuban Hospital, WWRC = Women’s Wellness and Research Center.

## Data Availability

Data and materials will be available from the corresponding author upon reasonable request, but may be subject to certain access restrictions due to ethical, legal, or commercial sensitivities.

## Public Involvement Statement

No public involvement in any aspect of this research.

## Guidelines and Standards Statement

This manuscript was drafted against the Strengthening the Reporting of Observational Studies (STROBE) guidelines for cross-sectional research [26].
